# Stability-Indicating RP-HPLC Method for the Simultaneous Estimation of Doxofylline and Terbutalinesulphate in Pharmaceutical Formulations

**DOI:** 10.3797/scipharm.1305-14

**Published:** 2013-07-14

**Authors:** Gananadhamu Samanthula, Krishnaveni Yadiki, Shantikumar Saladi, Sreekanth Gutala, K. V. Surendranath

**Affiliations:** 1 Department of Pharmaceutical Analysis, National Institute of Pharmaceutical Education and Research (NIPER), Balanagar, Hyderabad, India.; 2 United States Pharmacopeia – India Private Limited, Research and Development Laboratory, ICICI Knowledge Park, Turkapally, Shameerpet, Hyderabad, India.

**Keywords:** Doxofylline, Method Development, Chromatography, Stability, Terbutaline sulfate, Validation

## Abstract

An isocratic, stability-indicating, reversed-phase high-performance liquid chromatography (RP-HPLC) method was developed for the quantitative determination of doxofylline and terbutaline sulphate, used for the treatment of respiratory problems. The chromatographic separation was achieved on a Zorbax-SB Phenyl 250 × 4.6mm × 5 μm column with the mobile phase consisting of a mixture of 25 mM ammonium acetate (pH 5.0) : acetonitrile (85:15 %v/v) at a flow rate of 1.0 ml/min. The eluate was monitored at 274 nm using a PDA detector. Forced degradation studies were performed on the bulk sample of doxofylline and terbutaline sulphate using acid (0.1N HCl), base (0.1N NaOH), oxidation (10% hydrogen peroxide), photolytic, and thermal degradation conditions. Good resolution was observed between the degradants and analytes. Degradation products resulting from the stress studies did not interfere with the detection of doxofylline and terbutaline sulphate, thus the assay is stability-indicating. The method has the requisite accuracy, selectivity, sensitivity, and precision for the simultaneous estimation of doxofylline and terbutaline sulphate in bulk and pharmaceutical dosage forms. The limit of quantitation and limit of detection were found to be 1.16 μg/ml and 0.38 μg/ml for doxofylline, 2.08 μg/ml and 0.62 μg/ml for terbutaline sulphate, respectively.

## Introduction

**Fig. 1:**
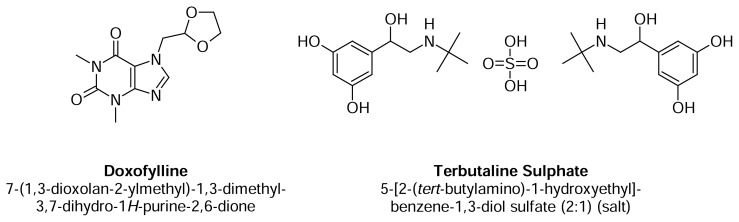
Structure of doxofylline and terbutaline sulphate.

A fixed dose combination of doxofylline and terbutaline sulphate is available for the treatment of asthma. Doxofylline and terbutaline sulphate chemical structures are given in Fig. 1.

Doxofylline is a new methyl xanthine derivative used in obstructive airway diseases and has similar efficacy as theophylline. But theophylline often results in a wide range of adverse effects, involving cardiac, GIT, and CNS, which accounts for the poor compliance and high dropout rates reported with its use. Moreover, it has a narrow therapeutic index, thus warranting strict monitoring of its level in the blood. Doxofylline has significantly fewer side effects, making the drug immensely beneficial to the patients [1–3]. Terbutaline sulphate is widely used as a bronchodilator for the treatment of bronchial asthma, chronic bronchitis, and emphysema. Terbutaline sulphate stimulates the α-adrenergic receptors of the sympathetic nervous system and has little or no effect on the adrenergic receptors [4, 5].

Recently, a fixed-dose combination of doxofylline and terbutaline sulphate was introduced in India [6]. Co-administration of doxofylline with terbutaline sulphate gives better bronchodilation with a lower degree of skeletal muscle tremor than a higher dose of terbutaline sulphate by mouth alone. Therefore, a fixed-dose combination of doxofylline and terbutaline sulphate is a better alternative for the treatment of acute and chronic asthma, as efficacy and safety goes hand-in-hand [7–11].

In the literature, several analytical methods were reported for the individual estimation of doxofylline [12–29] and terbutaline sulphate [30–33] in biological fluids and pharmaceutical formulations. Only three analytical methods were reported for the simultaneous estimation of doxofylline and terbutaline sulphate by spectrophotometry [34, 35] and recently by HPLC [36], but they are not stability-indicating. Hence, there is a need for developing a stability-indicating HPLC method for the simultaneous estimation of both drugs in pharmaceutical formulation. The present paper describes a simple, isocratic, stability-indicating HPLC assay method for the simultaneous quantification of doxofylline and terbutaline sulphate in pharmaceutical formulations.

## Experimental

### Chemicals and Reagents

Pure doxofylline was obtained from Mars Therapeutics Ltd, Secunderabad and pure terbutaline sulphate was obtained from Brundavan Laboratories, Hyderabad as a gift sample. Acetonitrile, methanol, formic acid, ammonium acetate, sodium hydroxide, hydrochloric acid, and hydrogen peroxide were purchased from Merck (Darmstadt, Germany). All reagents used were at least of analytical grade except acetonitrile and methanol, which was HPLC grade. HPLC grade water was obtained from a Millipore Milli-Q Plus system (Milford, MA, USA). All the standard and sample solutions were prepared in mobile phase. Tablet formulations, namely ZYLLINE-TR (Zubit life care) and PHYLEX-TR (Lexus) were purchased from a local market. The marketed formulations have a composition of 400 mg of doxofylline, 5 mg of terbutaline and excipients (q.s). The excipients may include hydroxypropyl methylcellulose, microcrystalline cellulose, anhydrous lactose, magnesium stearate, povidone, and pre-gelatinized starch.

### Instrumentation

Analysis was carried out using the Agilent 1100 series quaternary gradient HPLC with an autosampler and diode array detector (DAD). The Sartorius balance (CD 225 D, Germany) was used for weighing. The pH measurements were done on a pH-meter (Metrohm Schweiz AG, 780 pH meter, Germany) with an Epson printer Lx300t. A photostability chamber (Osworld, India) was used for the photo degradation study.

### Chromatographic Conditions

In preliminary experiments, the drugs were subjected to separation by using buffers like ammonium acetate (50 mM and 25 mM), acetonitrile, and methanol as organic modifiers at acidic pH on the C18 column. Good separation was observed on the Zorbax SB-Phenyl analytical column. Hence, the HPLC separation and quantification were made on the Zorbax SB-Phenyl analytical column (250 mm length, 4.6 mm i.d and 5 μm particle size). An isocratic mobile phase consisting of 25 mM ammonium acetate, pH-adjusted to 5.0 with 0.1% glacial acetic acid and acetonitrile in the proportion of 85:15% v/v at a temperature of 40 °C, were the final optimized method conditions. The eluate was monitored at 274 nm. The output signal was processed using Empower software of version 3.0.

### Method Validation

The method was validated for specificity, linearity and range, precision, accuracy, LOD and LOQ, robustness, and system suitability as per International Conference on Harmonization (ICH) guidelines [37].

#### Linearity and Range

ICH recommends a minimum of five concentrations over the concentration range of 80 to 120% of the test concentration for the assay method and 70 to 130% of the test concentration for content uniformity. Linearity was evaluated by analyzing seven concentrations of doxofylline and terbutaline sulphate making triplicate injections for each concentration. For linearity and range testing, stock solutions of doxofylline and terbutaline sulphate were prepared separately to contain 1 mg/ml of doxofylline and 0.1 mg/ml of terbutaline sulphate, respectively. Appropriate quantities of these stock solutions were mixed and diluted in a series of volumetric flasks to contain both the drugs in the concentration range of 280 to 520 μg/ml of doxofylline and 3.5 to 6.5 μg/ml of terbutaline sulphate, respectively (70 to 130% of the nominal concentration of both doxofylline and terbutaline sulphate present in tablet formulation). Linearity was checked for the assay method over the same concentration range for two consecutive days.

#### Specificity

The specificity of the developed HPLC method for the determination of doxofylline and terbutaline in bulk drug and pharmaceutical preparation (ZYLLINE-TR and PHYLEX-TR Tablets) was investigated by non-interference of placebo, forced degradation studies, and peak purity evaluation.

##### Non-Interference of Placebo

To check the non-interference of placebo, the placebo solution was prepared in the same way as that of the sample solution in the presence of all inactive ingredients of the tablet formulations, but without doxofylline and terbutaline.

##### Forced Degradation Studies

Excipients are usually inert substances and hence, these are not included in forced degradation studies. The forced degradation studies were done on drug substances and this was applied for drug product evaluation as per Dan W. Reynolds review [38]. The binary mixtures of doxofylline (1.5 mg/ml) and terbutaline sulphate (1.5 mg/ml) was subjected to hydrolytic and oxidative forced degradation studies. A 1:1 w/w solid mixture of doxofylline and terbutaline sulphate was used for thermal and photolytic degradation.

#### Hydrolysis

The hydrolytic degradation study of drugs was carried out in 0.1N HCl with reflux at 100°C for 5 hours (acid hydrolysis) and in 0.1N NaOH with reflux at 100°C for 5 hours (base hydrolysis). Finally, the resultant solutions were neutralized by adjusting pH to 7, this was done using 0.1 N HCl for base hydrolysis and 0.1 N NaOH for acid hydrolysis. The final concentration was adjusted to 0.15 mg/ml of each drug.

#### Oxidation

Binary mixtures of doxofylline and terbutaline sulphate were refluxed with 10% hydrogen peroxide (H_2_O_2_) for 5 hours at 80°C. The final concentration was brought up to 0.15 mg/ml of each drug.

#### Photo Degradation

Binary mixtures of both drugs were spread in 1 mm thickness in a petri dish under the exposure of 1.2 lux watt hours of UV light for 24 hrs in the photostability chamber. The sample solution was prepared to contain 0.15 mg/ml of each drug.

#### Thermal Degradation

Binary mixtures of both drugs were spread in 1 mm thickness in a petri dish and kept in a hot air oven for 48 hours at 80 °C. The mixture was dissolved in mobile phase to get 0.15 mg/ml of each drug.

#### Peak Purity Evaluation

The peak purity tool was used to check the peak purity of the drug and degradant peaks.

#### Precision Study

Repeatability was performed by analyzing six sample solutions prepared from the tablet formulation. Similarly, the intermediate precision was tested on two different days by two different analysts with the same tablet formulation.

#### Accuracy Study

The accuracy of the proposed method was demonstrated by preparing placebo samples spiked with 80%, 100%, and 120% of the test concentration of doxofylline and terbutaline sulphate present in the tablets. Each concentration level was prepared three times separately and analyzed. Mean % recovery and % RSD were calculated for each concentration. The ratio of the drug substance to placebo is 405 mg : 179 mg. For the placebo preparation, the excipients considered were hydroxypropyl methylcellulose (2 mg), microcrystalline cellulose (120 mg), anhydrous lactose (20 mg), magnesium stearate (2 mg), povidone (10 mg), and pre-gelatinized starch (25 mg). These amounts are used as per their normal ranges usually present in tablet formulations.

#### Limit of Detection (LOD) and Limit of Quantification (LOQ)

It was performed based on the signal-to-noise ratio. A standard solution of 5 μg/ml of doxofylline and 5 μg/ml of terbutaline sulphate solution was prepared to check the signal-to-noise ratios of the analytes. Then further dilutions were made for LOD and LOQ determination.

#### Robustness Study

The robustness test was performed by deliberately making the changes in the flow rate, buffer concentration, and pH of the mobile phase. Peak purity, retention time, tailing factor, resolution, and theoretical plates were measured to demonstrate the robustness of the method. Robustness was conducted on the sample solutions prepared from the tablet formulation.

#### System Suitability

Mixed standard solution of 400 μg/ml of doxofylline and 5 μg/ml of terbutaline sulphate solution was injected in six replicates and system suitability parameters were determined.

### Application to Analysis of Pharmaceutical Formulations

The proposed method was applied for the estimation of doxofylline and terbutaline sulphate in their tablet formulations. About twenty tablets were taken and pulverized to a fine powder, and then tablet powder equivalent to the average weight of one tablet was taken. The drugs were extracted with mobile phase for carrying out the analysis.

## Results and Discussion

### Results

**Fig. 2:**
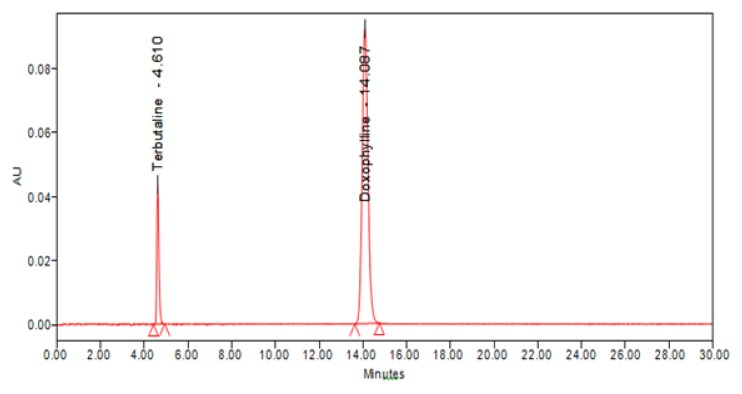
Typical chromatogram wherein the separation of doxofylline and terbutaline sulphate was shown using the method.

***An isocratic mobile*** phase consisting of 25 mM ammonium acetate of pH-adjusted to 5.0 with 0.1% glacial acetic acid and acetonitrile in the proportion of 85:15 %v/v was used in the present study. All determinations were performed at column temperature 40°C. The injection volume was 10 μL and the mobile phase was used as diluent for all sample preparations. The flow rate was 1.00 mL/min with UV detection at 274 nm. The typical chromatogram showing the separation of doxofylline and terbutaline sulphate was shown in Fig. 2. Doxofylline and terbutaline sulphate were eluted at a retention time of 4.61 and 14.08 min., respectively.

### Peak Purity Evaluation

Peak purity was determined by PDA, the results are shown in Tab 1.

The linearity for the proposed method was established by least squares regression analysis of the calibration curve. Calibration curves were linear over the concentration range of 280–480 μg/mL for doxofylline and 3.5–6.5 μg/mL for terbutaline sulphate with a correlation coefficient (r^2^) of 0.9997 ± 0.002 and 0.9997 ± 0.002, respectively. The precision of proposed method was good with a % RSD of below 1.0%. The results are presented in Tab. 2

Specificity was demonstrated by the placebo studies and through forced degradation studies. The non-interference of placebo is shown in Fig. 3.

Accuracy was checked by spiking the standard drugs doxofylline and terbutaline at three different concentration levels to the placebo. Recovery of individual components from the placebo ranged from 98.32 to 101.21%. Results are presented in Tab. 3.

The LOD of doxofylline and terbutaline sulphate was found to be 0.38 and 0.62 μg/mL and the LOQ of doxofylline and terbutaline sulphate was 1.16 and 2.08 μg/mL, respectively. In all the deliberate varied chromatographic conditions (flow rate, pH variation, buffer concentration), the system suitability parameters like tailing factor, resolution, and theoretical plates were not much affected, which shows that the method is robust. The results are shown in Tab. 4

#### Stability in Analytical Solution

***The solution stability for doxofylline and terbutaline was studied up to 48 hrs and the percentage peak area change observed was less than 1.0. Hence, the standard and sample solutions may be used up to 48 hrs after preparation.***

#### Analysis of Pharmaceutical Formulations

The proposed method was successfully applied to the assay of doxofylline and terbutaline sulphate in commercial tablets (ZYLLINE-TR and PHYLEX-TR). The percentage recoveries of both the drugs were based on the average of five replicate determinations (Tab. 5).

### Discussion

Stability-indicating assay methods are useful for determining the integrity of the drug substance and drug product during accelerated shelf life studies. It provides information about the drug quality. Therefore, there is a need for developing a stability-indicating HPLC method for the simultaneous estimation of doxofylline and terbutaline sulphate in pharmaceutical formulations. The proposed HPLC method was developed with an objective of separation of both the drugs and their degradants. Doxofylline is soluble in water and has a pKa of 9.87. Terbutaline sulphate is also soluble in water and has pKa values of 8.8, 10.64, and 11.1. As both drugs are moderately polar, reversed-phase HPLC was chosen as a separation mode. In the proposed method, a phenyl column was used as a phenyl-based reversed-phase as one of the first alternatives to C_18_ selectivity. They are compatible for polar compounds. Initially, trails were carried on C_18_ followed by C_8_, on these columns fronting was observed with early elution of doxofylline, so the method was opted with the phenyl column. The analysis was carried out at elevated temperature as high temperature favors good peak characteristics for both the drugs. Ammonium acetate was opted because of two reasons, MS compatibility and its pH range (3.8–5.8), which is within the desired pH of 5 for this analysis. From the results of the forced degradation studies, it can be concluded that doxofylline and terbutaline sulphate were stable under thermal stress conditions, but significant degradation was observed under acid and basic hydrolysis, and oxidative stress conditions. Slight degradation was observed under the photolytic stress conditions. From the peak purity test results and placebo studies, the purity threshold was greater than the purity angle; this confirms that the doxofylline and terbutaline sulphate peaks are homogeneous and pure in all the stress samples analyzed. The assay of doxofylline and terbutaline sulphate was unaffected by the presence of degradation products, thus confirming the stability-indicating power of the developed HPLC method. The forced degradation chromatograms are shown in Fig. 3a to 3e. Based on the results of accuracy, it can be concluded that the excipients used do not interfere in the analysis of doxofylline and terbutaline sulphate in their pharmaceutical formulations. Even the robustness study showed that the developed HPLC method was robust for the determination of doxofylline and terbutaline sulphate in pharmaceutical formulations within the selected ranges of chromatographic conditions.

## Conclusion

In the present work, a stability-indicating RP-HPLC method for the separation of doxofylline, terbutaline sulphate, and their degradants was developed and validated as per ICH guidelines. The proposed method is simple and effective. The proposed method is simple, effective, reliable, rugged, and suitable for the routine quality control of pharmaceutical formulations.

## Figures and Tables

**Fig. 3:**
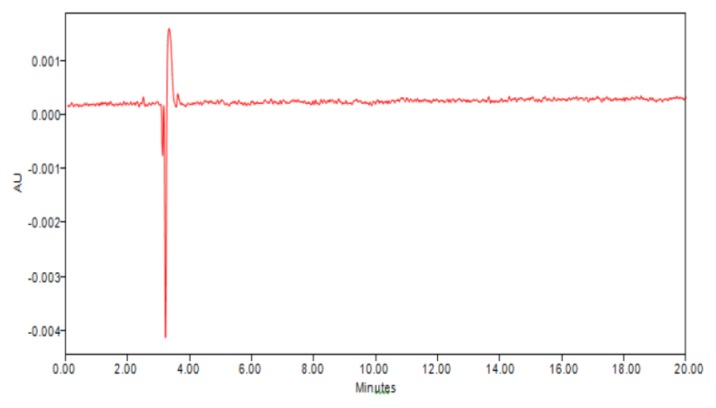
Chromatogram of placebo solution which shows non-interference of excipients.

**Fig. 3:**
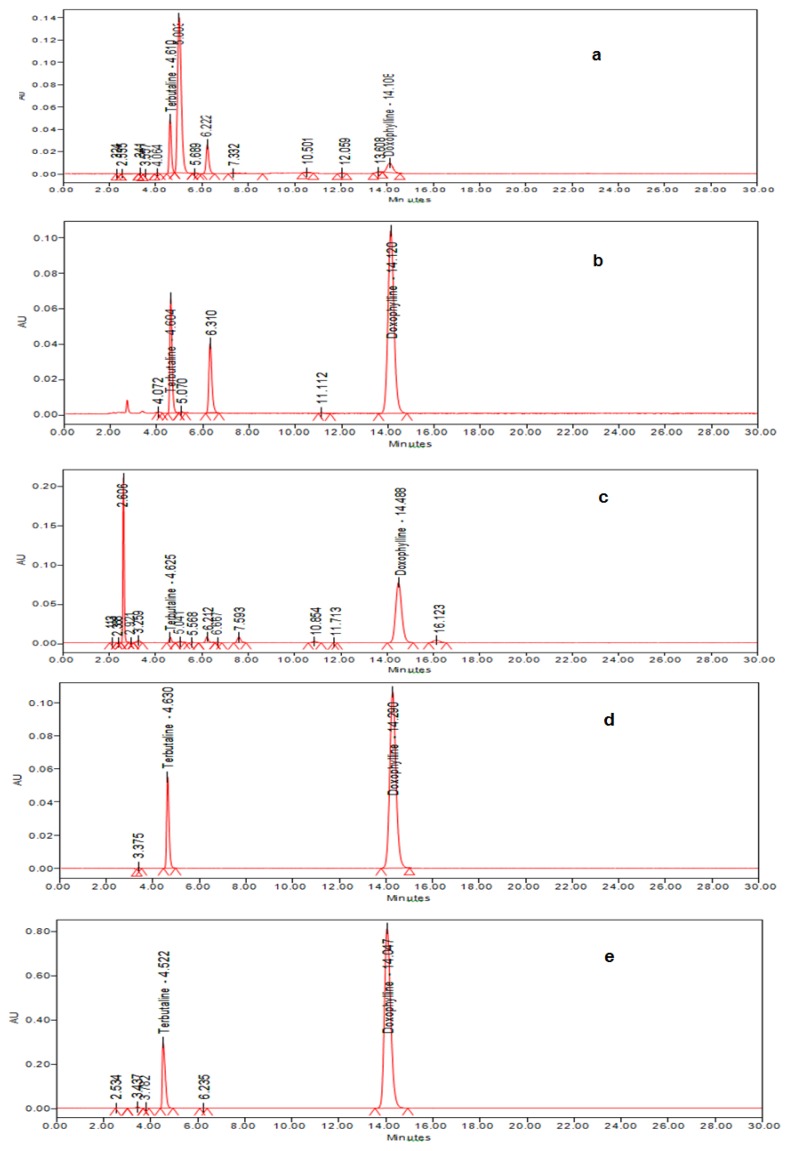
Chromatograms of the forced degradation study, which includes a) Acid stressed samples treated with 0.1 N HCl at 100°C for 5 hrs, b) Alkali stressed samples treated with 0.1N NaOH at 100°C for 5 hrs, c) Peroxide stressed samples treated with 10% H_2_O_2_ at 80 °C for 5 hrs, d) Photo stressed sample, e) Thermal stressed sample.

**Tab. 1:** Peak purity Data

Name	Purity angle	Purity threshold
Doxofylline	0.11	0.34
Terbutaline	0.22	0.51
Acid degrad. (Fig. 3a)		
Degradant 1	0.14	0.41
Degradant 2	1.29	1.32
Degradant 3	18.54	36.82
Degradant 4	15.92	30.36
Base degrad. (Fig. 3b)		
Degradant 1	39.72	90.0
Degradant 2	0.60	2.09
Degradant 3	54.37	90.0
Oxidative degrad. (Fig. 3c)		
Degradant 1	32.88	90.0
Degradant 2	56.82	90.0
Degradant 3	61.44	90.0
Degradant 4	10.52	90.0
Degradant 5	67.82	90.0
Photolytic degrad. (Fig. 3e)		
Degradant 1	36.66	68.05
Degradant 2	25.38	67.35

**Tab. 2:** Summary of validation parameters: Statistical data for the calibration graphs

Parameter	Doxofylline	Terbutaline
Linearity range	280–480 μg/ml	3.5–6.5 μg/ml
Correlation coefficient	0.9997 ± 0.002	0.9997 ± 0.002
Limit of detection	0.38 μg/ml	0.62 μg/ml
Limit of quantitation	1.16 μg/ml	2.08 μg/ml
Precision (%RSD)		
Intra-day (n=6)	0.03	0.04
Inter-day (n=6)	0.05	0.05
Analyst-1 (n=6)	0.04	0.06
Analyst-2 (n=6)	0.05	0.05

**Tab. 3:** Standard addition technique for determination of doxofylline and terbutaline by HPLC

Amount of drug added to placebo (mg)	Amount found (mg)	% Percentage Recovery	%RSD
a: Doxofylline			

280.11	279.89	99.92	1.25
400.37	396.33	98.99	1.54
480.22	486.03	101.21	1.23

b: Terbutaline			

3.54	3.55	100.28	1.64
5.12	5.09	99.41	1.34
6.56	6.45	98.32	1.29

**Tab. 4:** Results of robustness study

Description	Condition	Retention time (in min)		Tailing Factor		Resolution	Theoretical plate number	
	
		Dox	Ter	Dox	Ter		Dox	Ter
Flow rate	0.8	16.06	5.03	1.32	1.62	28.36	12564	6529
(mL/min)	1.2	11.67	3.78	1.2	1.68	25.61	12613	6422
Buffer	20	15.321	4.624	1.21	1.73	27.94	13285	7013
Conc.(mM)	30	14.011	4.386	1.21	1.57	27.75	13265	8160
pH of the	4.8	13.93	4.502	1.21	1.89	25.87	13383	6002
aqueous phase	5.2	13.722	4.297	1.21	1.398	28.27	13406	9200

Dox…Doxofylline; Ter…Terbutaline.

**Tab. 5:** Results of tablet analysis.

Formulation	Labelled amount (mg/tablet)		Amount found mg ± SD (n=5)		% Assay (n=5)	

	Dox	Ter	Dox	Ter	Dox	Ter
ZYLINE - TR	400	5	401.23 ± 1.25	5.01 ± 0.08	100.31	100.2
PHYLEX - TR	400	5	399.78 ± 1.78	5.13 ± 0.06	99.95	102.6

Dox…doxofylline; Ter…terbutaline sulphate.
